# Effects and mechanisms of prolongevity induced by *Lactobacillus gasseri* SBT2055 in *Caenorhabditis elegans*

**DOI:** 10.1111/acel.12431

**Published:** 2015-12-29

**Authors:** Hisako Nakagawa, Takuya Shiozaki, Eiji Kobatake, Tomohiro Hosoya, Tomohiro Moriya, Fumihiko Sakai, Hidenori Taru, Tadaaki Miyazaki

**Affiliations:** ^1^ Department of Probiotics ImmunologyInstitute for Genetic MedicineHokkaido UniversitySapporoJapan; ^2^ Milk science Research InstituteMegmilk Snow Brand Co., LtdKawagoeJapan; ^3^ Laboratory of Neuronal Cell BiologyGraduate School of Pharmaceutical SciencesHokkaido UniversitySapporoJapan

**Keywords:** aging, antioxidant, *Caenorhabditis elegans*, *Lactobacillus gasseri*SBT2055, longevity, mitochondria

## Abstract

Lactic‐acid bacteria are widely recognized beneficial host associated groups of the microbiota of humans and animals. Some lactic‐acid bacteria have the ability to extend the lifespan of the model animals. The mechanisms behind the probiotic effects of bacteria are not entirely understood. Recently, we reported the benefit effects of *Lactobacillus gasseri* SBT2055 (LG2055) on animal and human health, such as preventing influenza A virus, and augmentation of IgA production. Therefore, it was preconceived that LG2055 has the beneficial effects on longevity and/or aging. We examined the effects of LG2055 on lifespan and aging of *Caenorhabditis elegans* and analyzed the mechanism of prolongevity. Our results demonstrated that LG2055 has the beneficial effects on longevity and anti‐aging of *C. elegans*. Feeding with LG2055 upregulated the expression of the *skn‐1* gene and the target genes of SKN‐1, encoding the antioxidant proteins enhancing antioxidant defense responses. We found that feeding with LG2055 directly activated SKN‐1 activity via p38 MAPK pathway signaling. The oxidative stress response is elicited by mitochondrial dysfunction in aging, and we examined the influence of LG2055 feeding on the membrane potential of mitochondria. Here, the amounts of mitochondria were significantly increased by LG2055 feeding in comparison with the control. Our result suggests that feeding with LG2055 is effective to the extend lifespan in *C. elegans* by a strengthening of the resistance to oxidative stress and by stimulating the innate immune response signaling including p38MAPK signaling pathway and others.

## Introduction

Aging is a complex process characterized by a progressive impairment of the response of organisms to environmental stresses and general metabolic deterioration; however, altering certain processes can increase the lifespan. Hallmarks of aging include genomic instability, telomere attrition, epigenetic alteration, loss of proteostasis, deregulated nutrient sensing, mitochondrial dysfunction, and cellular senescence (López‐Otín *et al*., 2013). To date, many studies have focused on food sources, nutrients, and components that exert inhibitory effects on the hallmarks of aging in worms, flies, mice, and humans.

In 1907, Dr Metchnikoff first proposed the concept of probiotic bacteria, hypothesizing that lactobacilli were important for promoting human health and longevity (Metchnikoff, 1907) and that consumption of lactic‐acid‐producing bacteria (LAB), such as the lactobacilli found in yogurt, could enhance longevity. Many studies have reported the beneficial effects of LAB, including antimicrobial effects, microbial interference, and supplementary effects on nutrition (Rauch & Lynch, 2012).

*Caenorhabditis elegans* is possibly the most suitable model organism for research on aging. This is because it has an evolutionarily conserved metabolism and host defense mechanisms, including insulin/insulin‐like growth factor (IGF‐1) pathway, that is, the IIS pathway (Murphy *et al*., 2003), p38 mitogen‐activated protein kinase (p38 MAPK) pathway (Kim *et al*., 2002), and transforming growth factor‐β (TGF‐β) signaling pathway (Zugasti & Ewbank, 2009). Moreover, dietary resources, such as bacteria, play an important role in the control of the lifespan of *C. elegans* (So *et al*., 2011). In addition, probiotic bacteria provide functions that are important to the health and well‐being of the host and contribute in a number of ways to the functional improvement of foods (Wells & Mercenier, 2008). This is supported by reports indicating that specific LAB can enhance the host defense and stress resistance of *C. elegans* (Wang *et al*., 2011).

In this study, we focused on *Lactobacillus gasseri* SBT2055 (LG2055), which has been shown to exert beneficial effects in mice and humans, such as improvement of the intestinal environment, prevention of infection by influenza A virus (Nakayama *et al*., 2014) and augmentation of IgA production (Sakai *et al*., 2014), and lowering of the serum cholesterol concentration (Kajimoto *et al*., 2002). Therefore, it has been suggested that LG2055 exerts beneficial effects on longevity and/or aging.

It has also been suggested that LG2055 could exert beneficial effects on longevity and aging. Therefore, we investigated whether feeding with LG2055 extended the lifespan of and delayed aging in *C. elegans* and then analyzed the underlying mechanisms.

## Results

### LG2055 feeding extends *C. elegans* lifespan and increase mobility rate into the adult life stage

**Figure 1:**
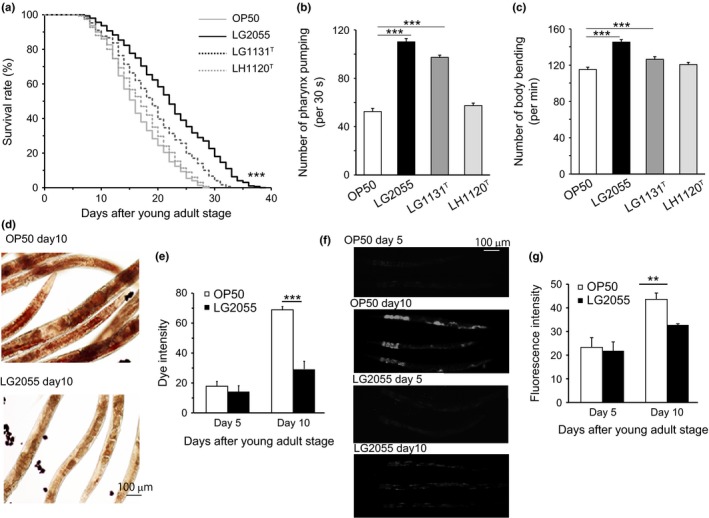
Effects of LG2055 feeding on the regulation of lifespan, motility, and expression of aging markers in *Caenorhabditis elegans*. (a) Effects of LG2055, *Lactobacillus gasseri* JCM1131^T^ (LG1131^T^) or *Lactobacillus helveticus* JCM1120^T^ (LH1120^T^) on the regulation of lifespan of the worms were determined. Control worms were fed with *Escherichia coli* OP50. Data are obtained by three independent experiments with 100 worms grown at 20 °C per group. Lifespan survival statistics are in Table S1. Pharynx pumping (b) or body bending (c) of the worms was counted after feeding with LG2055, LG1131^T^, or LH1120^T^ for 10 days after feeding stopped. Accumulation of lipid droplets was detected by oil red O staining (d), and dye intensity was measured (e) in the worms fed with LG2055 or OP50 for 5 or 10 days. Accumulation of lipofuscin was detected by autofluorescence (f), and fluorescence intensity was measured (g) in the worms fed with LG2055 or OP50 for 5 or 10 days. Results are shown by the SEM of three independent experiments with 10 worms in each group; statistically evaluated by an unpaired Student's *t*‐test (**P <* *0.01, and ***P <* *0.001).

We first examined whether feeding with LG2055 affected the lifespan of *C. elegans*. Age‐synchronous worms were grown to the young‐adult stage on nematode growth media plates seeded with *Escherichia coli* OP50, transferred individually to culture plates containing OP50 or LG2055, and then maintained at 20 °C. We observed that LG2055‐fed worms showed approximately a 37% increase in median survival, with a statistically significant right‐shifted survival curve (*P *<* *0.0001 by log‐rank test; Fig. 1a and Table S1). The presence of LG2055 in the guts of worms was confirmed by detecting fluorescein isothiocyanate (FITC)‐labeled LG2055 under a fluorescence microscope (Fig. S1a). We also tested lifespan with ultraviolet (UV)‐killed OP50 and LG2055 feeding. UV‐killed bacteria also extended worm lifespan to longer than was observed with live bacteria feeding, but the difference between groups did not change (Table S1). By changing the ratios of OP50 and LG2055, the lifespans of worms could be prolonged depending on the ratio of LG2055 (Fig. S1b). To evaluate the longevity effect of LG2055, we examined the effects of two *Lactobacillus* strains. *Lactobacillus gasseri* JCM1131^T^ (LG1131T)‐fed worms also exhibited a significant increase in lifespan, but the increase was lower than that observed in LG2055‐fed worms. Feeding the worms with *L. helveticus* JCM1120^T^ (LH1120T) had no significant effect on lifespan (Fig. 1a and Table S1). We also monitored the survival rate of *C. elegans* by changing the feeding ratio of LG2055 and OP50 and observed that lifespan was dependent on the concentration of LG2055 (Fig. S1b).

The locomotion rate is also considered closely correlated to longevity. Therefore, to investigate whether feeding with LG2055 increased the quality of life by extending the locomotory health span, we measured pharyngeal pumping and body bending rates. The pharyngeal pumping rate measures muscle function, and the pumping activity is associated with food intake ability. Although the frequency of pharyngeal pumping generally declined with age (Fig. S1c), it was two times higher in LG2055‐fed worms than in OP50‐fed worms on day 10 of adulthood (Fig. 1b). Next, we compared the body bending ability of the worms by counting the number of body bends per minute. The frequency of body bending declined with age (Fig. S1d), but the rate of decline was slower in the LG2055‐fed worms compared with the OP50‐fed worms. Similar to its effect on the survival rate, feeding with LH1120T did not exert any inhibitory effect on locomotion in *C. elegans* (Fig. 1b,c). Finally, feeding with LG2055 did not alter the brood egg numbers (Fig. S1 g). Overall, these results suggest that feeding with LG2055 effectively inhibited the age‐induced decrease in the mobility rate of *C. elegans*.

### Feeding with LG2055 inhibited the accumulation of lipid droplets and lipofuscin in worm bodies

Aging dysregulates intracellular lipid metabolism in several organisms. Therefore, we evaluated whether feeding with LG2055 affected lipid droplet accumulation with aging. Oil red O staining detected large amounts of intestinal fat in OP50‐fed worms, which was not observed in LG2055‐fed worms (Fig. 1d,e). Intracellular lipofuscin, an autofluorescent compound that accumulates in aging cells, is a marker of cellular damage during aging. Lipofuscin content also increases with aging in *C. elegans*; hence, we determined the levels in OP50‐ and LG2055‐fed worms on day 10 and 15 of adulthood. As expected, we observed that feeding with LG2055 delayed aging, with intestinal lipofuscin levels being 12% lower in LG2055‐fed worms than in OP50‐fed worms (Fig. 1f,g). Next, we examined senescence‐associated β‐galactosidase (SA‐β‐GAL) activity by performing β‐galactosidase staining and observed that SA‐β‐GAL activity increased in *C. elegans* with aging. However, the ratio of stained area in the whole body was decreased in LG2055‐fed worms compared with that in OP50‐fed worms (Fig. S1e,f). These results suggested that LG2055 feeding extended the lifespan of and decreased the rate of aging in *C. elegans*.

### Feeding with LG2055 enhanced the stress resistance of *C. elegans*

**Figure 2:**
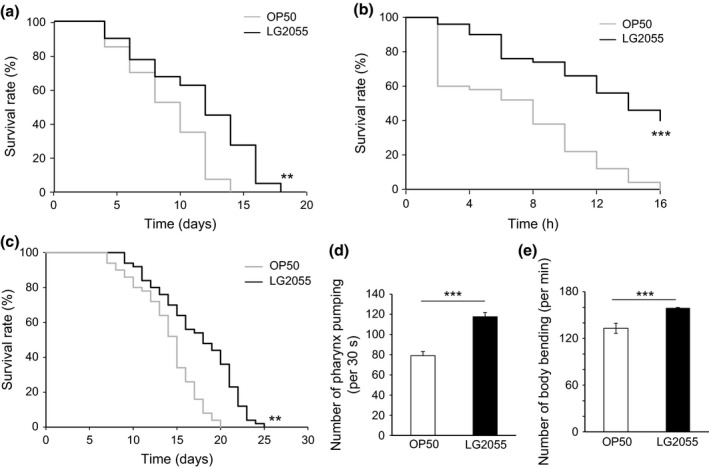
Effects of LG2055 feeding on the enhancement of stress resistance in *Caenorhabditis elegans*. (a) Survival rates of the worms by heat shock at 35 °C were determined after LG2055 or OP50 feeding for 10 days after feeding. The number of dead or alive worms was counted every day until all of OP50‐fed worms were dead. **P <* *0.01, log‐rank. (b) After the feeding of LG2055 or OP50 for 10 days, the worms were exposed to oxidative stress induced by paraquat. The number of dead or alive worms was counted every 2 h until all of OP50‐fed worms were dead, and the survival rates were calculated. ***P <* *0.001, log‐rank. (c) Effects of LG2055 on the regulation of lifespan of the *mev‐1* mutant worms. **P <* *0.01, log‐rank. The number of pharynx pumping (d) or body bending (e) of the *mev‐1‐*mutant worms was counted after feeding with LG2055 for 10 days after feeding. Results are shown by the SEM of three independent experiments with 10 worms in each group; statistically evaluated by an unpaired Student's *t*‐test. ***P <* *0.001,

In many studies of aging, extended longevity correlates with enhanced resistance against oxidative damage and other stresses. We analyzed the effect of feeding with LG2055 on thermal stress resistance in *C. elegans*. Thermotolerance of LG2055‐fed wild‐type worms was higher than that of OP50‐fed wild‐type worms (Fig. 2a and Table S1). The effect of feeding with LG2055 on oxidative stress resistance was also examined. After feeding with OP50 or LG2055 for 10 days, the worms were placed on agar supplemented with 25 mM of Paraquat. The number of dead or alive worms was counted every 2 h until all the OP50‐fed worms were dead. Approximately 40% OP50‐fed worms were dead 2 h after Paraquat treatment. In contrast, LG2055‐fed worms showed high resistance to oxidative stress and retained this resistance for 16 h after Paraquat treatment (Fig. 2b and Table S1). Methyl viologen‐sensitive gene (*mev‐1*) is a subunit of succinate coenzyme Q oxidoreductase that is a part of complex II of the electron transport system in the mitochondria. *C. elegans mev‐1* mutants show increased levels of intrinsic oxidative stress because of increased superoxide anion formation (Ishii *et al*., 1998), and we observed that feeding with LG2055 extended the lifespan of the *C. elegans mev‐1* mutant (Fig. 2c and Table S1). Further, feeding with LG2055 reduced the locomotion rate in the *mev‐1* mutant of *C. elegans* (Fig. 2d,e). These results suggest that the administration of LG2055 enhanced resistance against intrinsic oxidative stress, particularly in mitochondria, which slowed down the aging process.

### Feeding with LG2055 extended lifespan, but dependent on SKN‐1

**Figure 3:**
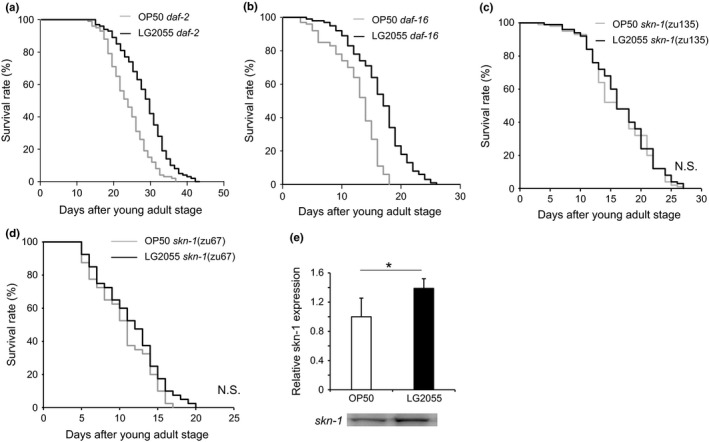
Regulation of genes expression in *Caenorhabditis elegans* by LG2055 feeding. (a–d) The survival rate of *daf‐2* (e1368), *daf‐16* (mgDf50), skn‐1 (*zu135*), and *skn‐1* (zu67) worms. Data were analyzed using log‐rank test. Lifespan survival statistics are in Table S2. (e) The difference of SKN‐1 protein level between feed type. *C. elegans* lysates quantitated by Western blotting with anti‐skn‐1 antibody. *P <* *0.05, Student's *t*‐test.

Next, we elucidated the pathway regulated by feeding with LG2055 for inducing its anti‐aging effect. In *C. elegans*, forkhead box protein O (FOXO) and SKN‐1 act in multiple longevity pathways. For example, the IIS pathway regulates the metabolism, development, and lifespan of *C. elegans*. Insulin‐like receptor (IR) is a transmembrane receptor that negatively regulates FOXO, which in turn promotes the expression of genes that confer extended longevity and enhance stress resistance (Lee *et al*., 2003; Murphy *et al*., 2003). To evaluate the involvement of IR and FOXO in the signaling pathway regulated by feeding with LG2055, we monitored the survival rates of *daf‐2* (e1368) and *daf‐16* (mgDf50) *C. elegans* mutants, respectively. We observed that feeding the *daf‐2* (e1368) and *daf‐16* (mgDf50) *C. elegans* mutants with LG2055 extended their lifespan similarly to that observed in wild‐type *C. elegans* (see Fig. 3a,b and Table S2 for details on these data and similar results from three other trials). Next, we looked for changes in the expressions of *daf‐2*,* age‐1*, and *daf‐16* after feeding with LG2055, but the expressions remained unchanged (Fig. S2a). We conclude that feeding with LG2055 can extend the lifespan via a mechanism that is independent of DAF‐2 and DAF‐16.

SKN‐1, an ortholog of mammalian NRF protein, is activated in response to oxidative stress or by compounds, such as hydrogen peroxide, Paraquat, and juglone, through the induction of phase II detoxification gene transcription (An & Blackwell, 2003). Tullet *et al*. (2008) indicated that the transcription network regulated by SKN‐1 promotes longevity and is an important direct target of IIS. SKN‐1 represents a connection between the development of the digestive system and one of its most basic functions – resistance to oxidative and xenobiotic stresses. The *skn‐1* mutant of *C. elegans* shows decreased resistance to oxidative stress and a shortened lifespan. In contrast, *C. elegans* mutants overexpressing SKN‐1, which constitutively localizes to the nuclei of intestinal cells, show increased resistance to oxidative stress and increased longevity (An & Blackwell, 2003; An *et al*., 2005; Tullet *et al*., 2008).

To investigate the role of SKN‐1 in LG2055‐induced lifespan extension in *C. elegans*, we examined the effects of feeding with LG2055 with two independent *skn‐1* alleles. The chosen alleles were *skn‐1* (zu135), which contains a premature stop codon in the SKN‐1 DNA‐binding domain, and *skn‐1* (zu67), which lacks the regions encoding the SKN‐1A and SKN‐1C isoforms (Bowerman *et al*., 1992; Bishop & Guarente, 2007; Johns *et al*., 2007; Tullet *et al*., 2008). We did not confirm that *zu67* produces functional SKN‐1b/c and that this stop codon mutation within the other isoforms does not affect b/c transcription. SKN‐1 encodes three protein isoforms, namely, SKN‐1A, SKN‐1B, and SKN‐1C, which have different aminoterminal domains but similar carboxyl‐terminal domains. SKN‐1 is present in nuclei constitutively in the ASI neurons (putative hypothalamus) (An & Blackwell, 2003), where it is required for longevity to be extended by dietary restriction (DR), a condition that increases longevity in organisms as diverse as yeast and rodents (Bishop & Guarente, 2007). The stress resistance function of SKN‐1 is mediated by its expression in the intestine (digestive system) (Bishop & Guarente, 2007), where SKN‐1 accumulates in nuclei and activates phase 2 gene expression inducibly in response to stress (An & Blackwell, 2003). We observed that feeding with LG2055 no longer conferred a median lifespan of *C. elegans* mutants carrying the *skn‐1* alleles (trials repeated in triplicate per strain; Fig. 3c,d and Table S2). Because the longevity effect of feeding with LG2055 was related to SKN‐1 activation, we examined the effects of feeding with LG2055 on SKN‐1 expression in the intestines using LG349 geIs10 (*ges‐1p[long]::skn‐1 c*::GFP + rol‐6 [su1006]) (Bishop & Guarente, 2007). We observed that extrachromosomal and integrated transgenes driving *skn‐1c–gfp* expression from *ges‐1* promoter 16 induced stronger fluorescence in LG2055‐fed worms than in OP50‐fed worms (Fig. S2b). These results suggest that feeding with LG2055 induced the nuclear accumulation of SKN‐1‐GFP in the intestine. Thus, these data indicated that SKN‐1A, SKN‐1C, or both functioned in the intestine of *C. elegans* to mediate the effects of LG2055‐induced longevity.

### Feeding with LG2055 directly activated SKN‐1 activity via p38 MAPK pathway signaling

Next we considered how LG2055‐activated SKN‐1 exerted upstream effects to confer health span benefit. The stress resistance function of SKN‐1 is known to be mediated by its expression in the intestines (Bishop & Guarente, 2007) where SKN‐1 accumulates in nuclei and activates the inducibility of phase 2 gene expression in response to stress (An & Blackwell, 2003). In the intestine, mitogen‐activated protein kinase (PMK‐1) phosphorylates SKN‐1, which then translocates to the nuclei of intestinal cells and induces the transcription of phase 2 detoxification genes (Inoue *et al*., 2005). We observed that feeding with LG2055 altered the expression of genes involved in p38 MAPK signaling (Fig. S2a). The transcriptional activity of SKN‐1 is regulated by phosphorylation by PMK‐1, a major regulator of innate immunity in *C. elegans*; therefore, the loss of PMK‐1 induced a strong susceptibility phenotype. Analysis of PMK‐1 showed that it was regulated by a phosphorylation cascade involving the activation of sterile alpha and TIR motif‐containing protein (TIR‐1) that activated mitogen‐activated protein kinase kinase kinase (NSY‐1), which in turn activated dual specificity mitogen‐activated protein kinase kinase (SEK‐1) and then induced PMK‐1 phosphorylation.

**Figure 4:**
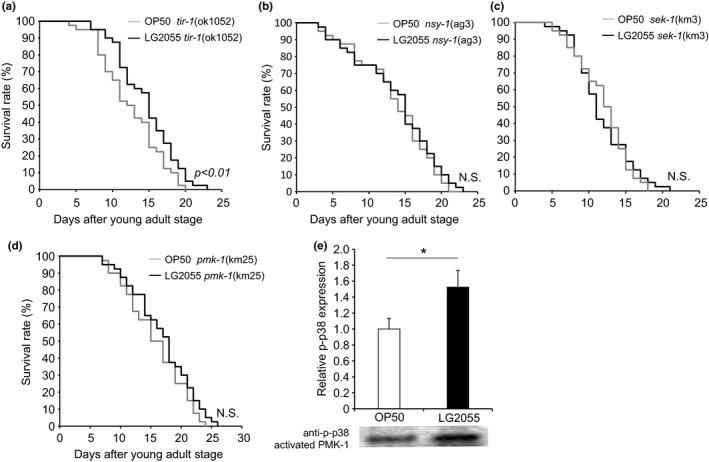
Effects of LG2055 feeding on the *Caenorhabditis elegans* p38 MAPK pathway. (a–d) Lifespan analysis of *tir‐1* (ok1052), *nsy‐1* (ag3), *sek‐1* (km3), and *pmk‐1* (km25). Data are obtained by three independent experiments with 100 worms grown at 20 °C per group. Lifespan survival statistics are in Table S2. (e) Activation of PMK‐1 by LG2055 feeding. After the feeding of LG2055 or OP50 for 10 days, extracts prepared from each animal were immunoblotted (IB) with antiphospho p38. *P <* *0.05, Student's *t*‐test.

SEK‐1 is also known to be required for the activation of SKN‐1 during oxidative stress. To investigate whether feeding with LG2055 activated the p38 MAPK pathway, we performed survival assays on three occasions, and each produced similar results. Feeding with LG2055 marginally increased the lifespan of the *tir‐1* mutant of *C. elegans* (Fig. 4a and Table S2) but did not increase the mean lifespan of the *nsy‐1*,* sek‐1*, or *pmk‐1* mutants (Fig. 4b–d and Table S2). In addition, levels of phosphorylated p38 were increased in LG2055‐fed worms (Fig. 4e). Thus, our results indicated that the p38 MAPK pathway contributed to LG2055‐induced longevity.

### Feeding with LG2055 increases the mRNA expression of stress resistance genes

**Figure 5:**
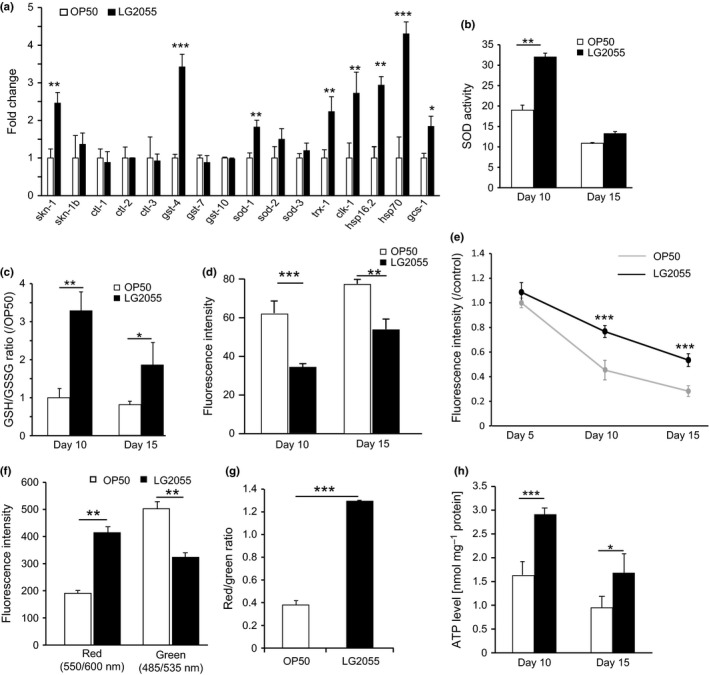
Critical responses to oxidative stress regulated by LG2055. (a) The mRNA expression change of genes, *skn‐1, skn‐1*‐regulated genes, and oxidative stress resistance genes by LG2055 feeding was evaluated by the real‐time PCR. mRNA levels were normalized with that of *act‐1* as a control; error bars represent SEM. *P <* *0.05, ** *P *<* *0.01, and *** *P *<* *0.001 statistically evaluated by an unpaired Student's *t*‐test. (b) The effect of LG2055 feeding on SOD activity in the body of worms. (c) The effect of LG2055 feeding on GSH/GSSG ratio in the worms. The effect of LG2055 feeding on the accumulation of ROS measured by fluorescence intensity in N2 WT. (d) Results are shown as the mean±SE by three independent experiments with 20 worms per each group; statistically evaluated by an unpaired Student's *t*‐test (**P <* *0.01, and ***P <* *0.001). (e) Mitochondria in the worm cells after OP50 or LG2055 feeding were stained using Mitotracker Red CM ROS. The fluorescence intensity of the stained mitochondria was measured. (f) In worms 10 days from young adult, red or green fluorescence intensity by JC‐1 staining was measured after OP50 or LG2055 feeding. (g) 10 randomly selected worms by each feeding group were examined for the measurement of the ratio of red/green fluorescence intensity by JC‐1 staining. (h) ATP levels in the whole body of the worms after LG2055 and OP50 feeding. Differences compared with OP50‐fed worms were considered statically significant when *P <* *0.05, **P <* *0.01, and ***P <* *0.001 by Student's *t*‐test.

Antioxidant defenses can be altered by genetic. To examine whether feeding with LG2055 affected the expression SKN‐1‐controlled and age‐related genes, including those encoding catalases (*ctl‐1*,* ctl‐2*,* and ctl‐3*), superoxide dismutases (SODs) (*sod‐1*,* sod‐2*, and *sod‐3*), and several glutathione *S*‐transferases (GSTs; *gst‐4*,* gst‐7*, and *gst‐10*), we performed real‐time PCR to quantitate the mRNA levels of these genes. We observed significantly increased expressions of *skn‐1*,* gst‐4*,* sod‐1*,* trx‐1* (thioredoxin), *clk‐1* (mitochondrial polypeptide), *hsp16.2* (heat‐shock protein), *hsp‐70*, and *gcs‐1* (an ortholog of γ‐glutamyl‐cysteine synthetase) in the LG2055‐fed worms (Fig. 5a). Next, we analyzed the changes in the expression of SKN‐1 target genes of *skn‐1* (zu135) after feeding with LG2055; however, feeding with LG2055 did not change the expression levels of these genes (Fig. S2c).

### LG2055 decreases reactive oxygen species by enhancing superoxide dismutase activity and increasing glutathione/glutathione disulfide

Based on the results of upregulated expression of stress resistance genes, we examined the effect of feeding with LG2055 on SOD activity and the glutathione/glutathione disulfide (GSH/GSSG) ratio. Compared with OP50‐fed worms, the LG2055‐fed worms showed an increase of approximately 80% in SOD activity on day 10 of adulthood. However, this difference was very small on day 15 of adulthood (Fig. 5b).

Under normal physiological conditions, most of the GSH is present in the reduced form, and only a small fraction is present as GSSG (Li *et al*. 2004). Because oxidative stress induces the oxidation of GSH to GSSG, the GSH/GSSG ratio is an indicator of the cellular redox state and reflects antioxidative capacity. We observed that the GSH/GSSG ratio increased threefold in the LG2055‐fed worms compared with the OP50‐fed worms (Fig. 5c).

GSH is a functional scavenger of reactive oxygen species (ROS), including superoxide anions, hydrogen peroxide, and hydroxyl radicals. Superoxide anions are typically the primary ROS and are generated by the action of dihydronicotinamide adenine dinucleotide phosphate (NADPH) oxidase on oxygen molecules. Therefore, we measured the superoxide anion production using dihydroethidium (DHE), which is converted to a fluorescent compound upon oxidation by superoxide anions. We observed that the ROS level increases with age. However, ROS accumulation showed a strong decrease in fluorescence intensity in LG2055‐fed worms (Fig. 5d), suggesting that LG2055 inhibited ROS accumulation in *C. elegans*.

### LG2055 reduces the mitochondrial function declines with aging

The mitochondria are the major site of ROS production in cells, which is controlled by the mitochondrial membrane potential. Therefore, we used a MitoTracker^®^ CMXRos to determine whether feeding with LG2055 affected mitochondrial functioning. The MitoTracker^®^ works by staining the mitochondria in live cells, and its accumulation is dependent upon membrane potential. Mitochondrial membrane potential was significantly higher in LG2055‐fed aged worms than in OP50‐fed aged worms (Fig. 5e). In addition, we analyzed the effect of feeding with LG2055 on mitochondrial membrane potential using cyanine dye JC‐1, which is widely used to assess the electrical gradient across the mitochondrial inner membrane (ΔΨm) because it is more specific for this than it is for the plasma membrane potential. Apoptotic cells show green fluorescence because JC‐1 is present in its monomeric form in these cells, whereas nonapoptotic cells show red fluorescence because of the aggregation of JC‐1 in the mitochondria. LG2055‐fed worms showed higher red fluorescence intensity and lower green fluorescence intensity on day 10 of the young‐adult stage (Fig. 5f). The ratio of red/green fluorescence intensity was significantly higher in LG2055‐fed worms (Fig. 5g).

Given that an important function of the mitochondria is to produce chemical energy [i.e., adenosine triphosphate (ATP)], we also determined whether feeding with LG2055 affected ATP levels in *C. elegans*. ATP levels were measured by performing a bioluminescence assay after feeding the worms with OP50 or LG2055 for 10 and 15 days. ATP levels decrease gradually with aging. However, we observed that ATP levels were higher in LG2055‐fed worms than in OP50‐fed worms (Fig. 5h). These data suggest that feeding with LG2055 effectively inhibited age‐dependent impairment of mitochondrial functioning.

## Discussion

Probiotics have been defined as ‘live microorganisms, which when administered in adequate amounts, confer a beneficial health effect on the host’. However, many of the effects obtained from viable cells of probiotics are also obtained from populations of dead cells (Adams, 2010) such as induction of B‐cell activation, induction of TNF‐α production, and augmentation of IgA production. In the human gastrointestinal tract, LG2055 establishes and alters the composition and metabolism of the intestinal microflora as well as the physical characteristics of the feces (Fujiwara *et al*., 2001). Dead LG2055 has also been shown to provide some health benefits in both mice and humans, and although LG2055 was not shown to be harmful, it was unable to colonize the intestines of *C. elegans*. In the first part of this study, we showed that when compared with OP50 feeding, feeding both live and dead LG2055 increased the lifespan. Given that the administration of dead LG2055 remained beneficiary, LG2055 cannot be considered probiotics based on the classic definition (Sanders *et al*., 2007). However, it is certain that LG2055 is the beneficial bacteria. It therefore seems likely that the functional molecule is not a microbial metabolite. Next, we investigated the longevity mechanism of LG2055.

**Figure 6:**
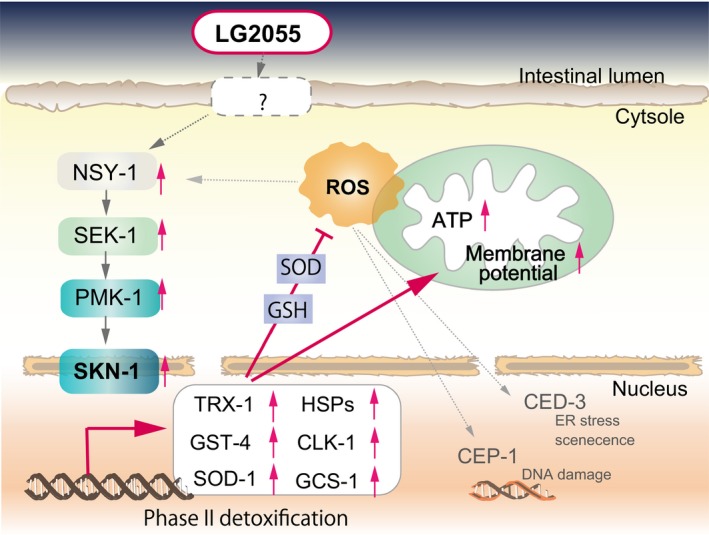
Schematic representation of signaling pathways regulated by LG2055 feeding in *Caenorhabditis elegans*. LG2055 feeding induces the activation NSY‐1–SEK‐1–PMK‐1 and upregulates the expression of SKN‐1 and its target genes. Then, induced antioxidant molecules enhances the antioxidant defense response and prevents reduction of mitochondrial functions such as ATP production and increasing membrane potential. Therefore, LG2055 feeding enhances the stress resistance.

Aging is a complex process driven by diverse molecular signaling pathways, and to date, three anti‐aging pathways have been established (Fig. 6). Many genes that are differentially regulated in young vs. old animals are known or postulated to be regulated by DAF‐16 and SKN‐1. DAF‐16 and SKN‐1 transcription factors play highly conserved roles in regulating stress resistance and longevity genes.

SKN‐1 plays physiological regulatory roles in multiple processes, including detoxification, metabolism, and the immune response. We observed that feeding with LG2055 did not extend the lifespan of *skn‐1* mutant worms (Fig. 3c,d and Table S2). However, we did not confirm the difference in SKN‐1 protein levels between OP50‐ and LG2055‐fed worms (Fig. 3e). SKN‐1 encodes three isoforms, namely, SKN‐1A, SKN‐1B, and SKN‐1C, which have different aminoterminal domains but similar carboxyl‐terminal domains (Bishop & Guarente, 2007). Bishop & Guarente (2007) suggested that SKN‐1C induced resistance to oxidative stress in the intestines. In our results, similar to oxidative stress, the intensity of the fluorescence for SKN‐1 in the gut was significantly stronger in LG2055‐fed *C. elegans* (LG349 geIs10) than in OP50‐fed controls (Fig. S2b).

The maintenance of low ROS levels is critical to normal cell function. Thus, we also investigated whether LG2055 stimulated the host defense system and ROS production. Hoeven *et al*. (2011) have shown that ROS released from Ce‐Duox1/BLI‐3 can activate SKN‐1 activity via p38 MAPK signaling, with NSY‐1 and SEK‐1 both able to regulate the p38 MAPK ortholog PMK‐1. In response to oxidative stress, PMK‐1 phosphorylates SKN‐1, which then translocates to the nuclei of intestinal cells and induces the transcription of phase 2 detoxification genes (Inoue *et al*., 2005). The p38 MAPK pathway is also known to be crucial for stress response and immunity. Papp *et al*. (2012) showed that SKN‐1 and PMK‐1 are central elements in immunosenescence. Immunosenescence, or the age‐dependent decline in immune responsivity, is a critical condition that impedes healthy aging (Aw *et al*., 2007). Therefore, we hypothesize that LG2055 inhibits the accumulation of oxidative damage associated with aging by stimulating the immune system, including p38 MAPK signaling and other pathways. Feeding with LG2055 marginally increased the lifespan of the *tir‐1* mutant of *C. elegans* (Fig. 4a and Table S2) but did not increase the mean lifespan of the *nsy‐1*,* sek‐1*, or *pmk‐1* mutants (Fig. 4b–d and Table S2). We conclude that feeding with LG2055 effectively stimulated p38 MAPK signaling.

Simultaneously, we proved that feeding with LG2055 activated downstream *skn‐1* genes. Previous studies have shown that SKN‐1 activation induces the transcription of antioxidant genes, while in this study, we showed that feeding with LG2055 significantly increased the expression of *sod‐1*,* gst‐4*,* trx‐1*,* gcs‐1*,* clk‐1*,* hsp16.2*, and *hsp‐70* (Fig. 5a). Expression of *sod‐1* and the genes encoding heat‐shock proteins is induced in the presence of oxidative stress (Park *et al*., 2009), with SOD‐1 contributing to 80% of the total SOD activity in *C. elegans* (Doonan *et al*., 2008). We observed that SOD activity was increased in LG2055‐fed worms compared with that in OP50‐fed worms (Fig. 5b). GSTs detoxify reactive compounds by conjugating reduced GSH to electrophilic centers. Oxidative stress induces the expression of *gcs‐1*, which encodes γ‐glutamyl‐cysteine synthetase, a rate‐limiting enzyme in GSH synthesis. GSH plays a central role in redox regulation, augmentation of ROS defense, and phase II detoxification. In the present study, feeding with LG2055 increased the GSH/GSSG ratio (Fig. 5c) and decreased ROS levels (Fig. 5d).

The results that ROS accumulation were decreased, SODs were activated, the lifespan of the *mev‐1* mutant of *C. elegans* was prolonged, and the expression of genes encoding heat‐shock proteins were increased by feeding with LG2055 strongly suggest regulation of mitochondrial functioning. Mitochondrial functions are associated with aging in several ways, and the magnitude of these functions declines with age. Defects in mitochondrial homeostasis are closely linked to the development of human pathologies such as cancer, type 2 diabetes, and additional metabolic disorders (Wallace, 2005). While limited amounts of ROS appear to exert health‐promoting functions in diverse species (Ristow & Zarse, 2010), mitochondrial dysfunction and protein damage are mainly caused by excessive formation of ROS, which are by products of oxidative phosphorylation and energy production in the form of ATP (Tatsuta, 2009; Segref *et al*., 2014). We observed that feeding with LG2055 increased the amounts of mitochondria (Fig. 5e) and increased the mitochondrial membrane potential (Fig. 5f,g), which is the central bioenergetic parameter that controls respiration rate and ATP synthesis (Nicholls, 2004). In the last part of this study, we confirmed that feeding with LG2055 increased ATP levels (Fig. 5h), while ATP levels declined with age in the OP50‐fed worms.

Our results suggested that P38 MAPK signaling stimulated LG2055 to produce one of anti‐aging effect. Ikeda *et al*. (2007) have also studied the effects of different probiotic strains in *C. elegans*, including *Bifidobacterium*,* Lactobacillus helveticus*, and *Lactobacillus plantarum*. Immune‐stimulating molecules, such as peptidoglycan (Lebeer *et al*., 2010), S‐layer protein (Konstantinov *et al*., 2008), and exopolysaccharide (Kim & Kim, 2009; Kim *et al*., 2010) exist on the cell surfaces of these bacteria. However, our study showed that *Lactobacillus helveticus* did not affect longevity in *C. elegans* and that the effect of a *L. gasseri* type strain was weak compared with that of LG2055. Therefore, the beneficial efficacy of LAB may be influenced by differences in the structures of immune‐stimulating molecules. Equally, some other as yet unknown factor could be involved.

In conclusion, we showed that feeding with LG2055 effectively extended the lifespan of *C. elegans* by increasing stress resistance and stimulating the immune response (Fig. 6).

## Experimental procedures

### *Caenorhabditis elegans* maintenance

We used the following strains: the wild‐type N2, Bristol (wild‐type); GR1307, *daf‐16* (mgDf50); DR1572, *daf‐2* (e1368); TK22, *mev‐1* (kn1); EU1, *skn‐1* (zu67)/nT1[unc‐?(n754);let‐?]; EU31, *skn‐1* (zu135)/nT1[unc‐?(n754);let‐?]; AU3, *nsy‐1* (ag3) II; KU4, *sek‐1* (km 4)X; KU25, *pmk‐1* (km25)IV; RB1085, *tir‐1* (ok1052) III; LG349 geIs10 [ges‐1p (long)::skn‐1c::GFP + rol‐6 (su1006)) strains. All nematodes were grown and maintained at 20 °C on nematode growth medium (Muller *et al*., 2007) plates seeded with *E. coli* OP50 (Brenner 1974).

### Lifespan measurement

Lifespan measurement were performed in 4‐cm tissue culture plates, with each plate containing 7.5 μM 5‐fluorouracil. Age‐synchronous worms were growth to the L4/young‐adult stage on NGM plates seeded with OP50 then transferred individually into the OP50 or LG2055 plates and maintained at 20 °C. Thereafter, worms were transferred every 2–3 days until all worms were dead. Dead worms were scored daily and removed immediately. An animal was scored as dead when it did not respond to mechanical stimulation. Survival analysis was performed using the Kaplan–Meier method on censored data, and the significance of differences between survival curves was calculated using the log‐rank test. The statistical software used was EZR (Saitama Medical Center, Jichi Medical University), which also computed the median lifespan. More than 100 animals per group were analyzed in each experiment, and at least three independent replications were performed for each assay.

### Other methods

Preparation of LG2055 cells, Ingestion of FITC‐labeled LG2055, age synchronization, motility assays, age pigment (lipofuscin) accumulation, oil red O staining, SA‐β‐GAL activity assay, thermotolerance, oxidative stress assay, *skn‐1*:GFP localization assay, RNA preparation and Q‐PCR analysis, Western blot analysis, SOD activity assays, GSH/GSSG assay, DHE staining, mitochondria staining, JC‐1 staining, and measurement of ATP levels are described in the Supporting experimental procedures.

## Author contributions

H.N., T.S., and T. Miyazaki conceived and planned this study. H.N. performed the research and analyzed all data. T.S. set up the experimental tool. All the authors discussed the results and commented on the manuscript. H.N. and T. Miyazaki wrote the manuscript.

## Funding

No funding information provided.

## Conflict of interest

E.K., T.H., T. Moriya, and F.S. are employees of Megmilk Snow Brand Co., Ltd. H.N., T.S., and T. Miyazaki declared no conflict of interest.

## Supporting information


**Fig. S1** LG2055 feeding extended the lifespan and slowed the aging speed in *Caenorhabditis elegans*. (a) Photomicrograph of the *C. elegans* after feeding of FITC‐labeled LG2055. (b) Survival curves of *C. elegans* fed with OP50 mixed with LG2055 at different ratios from 0 to 100%. The number of pharynx pumping (c) and body bending (d) was measured on day 5 and 10 after OP50 or LG2055 feeding. (e,f) Photomicrograph of the *C. elegans* stained by the senescent‐associated β‐galactosidase (SA‐β‐Gal+) on day 15 after OP50 or LG2055 feeding. Results are shown by the SEM of three independent experiments with 10 worms in each group; statistically evaluated by an unpaired Student's *t*‐test (*P <* *0.05). (g) Numbers of eggs laid by LG2055‐ and OP50‐fed worms were determined on day 1–5 after OP50 or LG2055 feeding. Results are shown by the SEM of three independent experiments with 10 worms in each group; statistically evaluated by an unpaired Student's *t*‐test (*P <* *0.05, **P <* *0.01, and ***P <* *0.001).

**Fig. S2** LG2055 feeding changed the gene expression of the molecules in p38MAPK signaling cascades. (a) Expression of the mRNA levels of the IIs pathway‐related genes, *let‐363*, and p38MAPK signaling molecules, on adult day 10 after OP50 or LG2055 feeding was measured by real‐time PCR. Expression levels of mRNA were normalized to the *act‐1* mRNA expression level; error bars represent SEM. Differences compared with OP50‐fed worms were considered statically significant as *P <* *0.05, *P <* *0.01, and ***P <* *0.001 by Student's *t*‐test. (b) Expression of *skn‐1c* was determined by fluorescence of GFP in the worms expressed from the gels10 transgenes after LG2055 or OP50 feeding for 10 days after feeding. (c) Expression level of SKN‐1 in the intestine of the worms was compared between LG2055 and OP50 feeding by the measurement of fluorescence intensity. Results are shown by the SEM of three independent experiments with 10 worms in each group; statistically evaluated by an unpaired Student's *t*‐test (*P <* *0.05).

**Table S1** The effects of environmental variables upon lifespan in the presence or absence of LG2055.

**Table S2** The effects of genetic variables upon lifespan in the presence or absence of LG2055.

**Table S3** Primer sets used for quantitative RT‐PCR.

**Appendix** S1 Supporting experimental procedures.
