# Supplementary figures and images for: Effects and mechanisms of prolongevity induced by Lactobacillus gasseri SBT2055 in Caenorhabditis elegans
Source: Aging Cell. 2015 Dec 29;15(2):227–36. doi: 10.1111/acel.12431 (PMC4783334; doi:10.1111/acel.12431)

a)

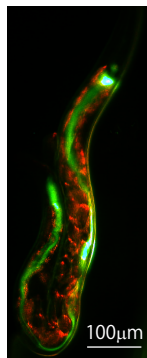

b)

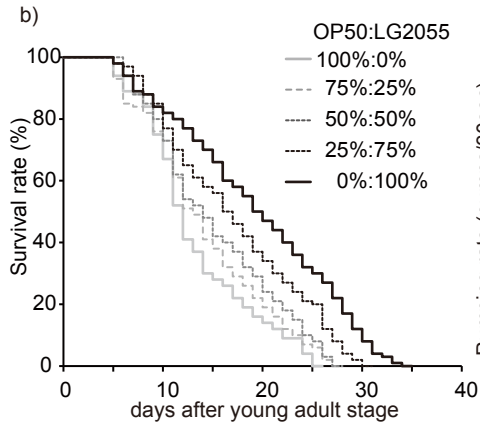

c)

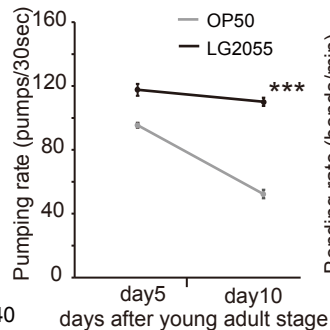

d)

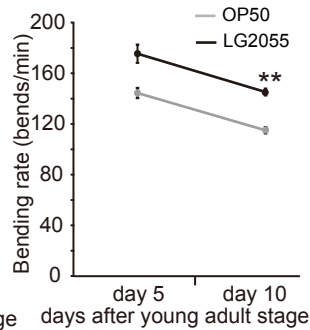

e)

f)

OP50 day15

LG2055 day15

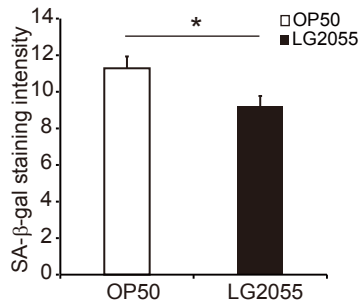

g)

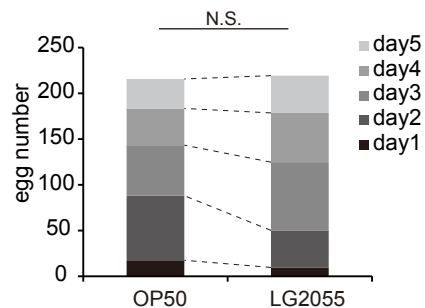

100 μm

Supplement: Supplementary file 1 — Fig. S1 LG2055 feeding extended the lifespan and slowed the aging speed in Caenorhabditis elegans. (a) Photomicrograph of the C. elegans after feeding of FITC‐labeled LG2055. (b) Survival curves of C. elegans fed with OP50 mixed with LG2055 at different ratios from 0 to 100%. The number of pharynx pumping (c) and body bending (d) was measured on day 5 and 10 after OP50 or LG2055 feeding. (e,f) Photomicrograph of the C. elegans stained by the senescent‐associated β‐galactosidase (SA‐β‐Gal+) on day 15 after OP50 or LG2055 feeding. Results are shown by the SEM of three independent experiments with 10 worms in each group; statistically evaluated by an unpaired Student's t‐test (*P < 0.05). (g) Numbers of eggs laid by LG2055‐ and OP50‐fed worms were determined on day 1–5 after OP50 or LG2055 feeding. Results are shown by the SEM of three independent experiments with 10 worms in each group; statistically evaluated by an unpaired Student's t‐test (*P < 0.05, **P < 0.01, and ***P < 0.001). [file ACEL-15-227-s001.pdf]

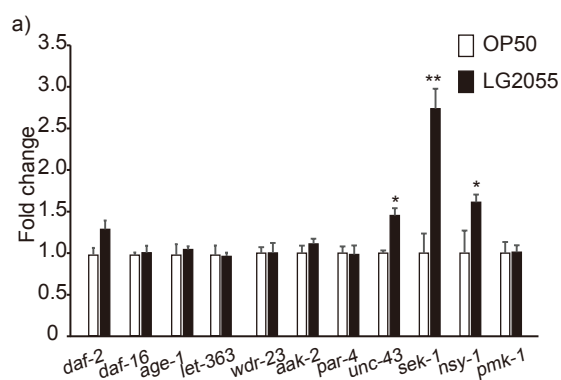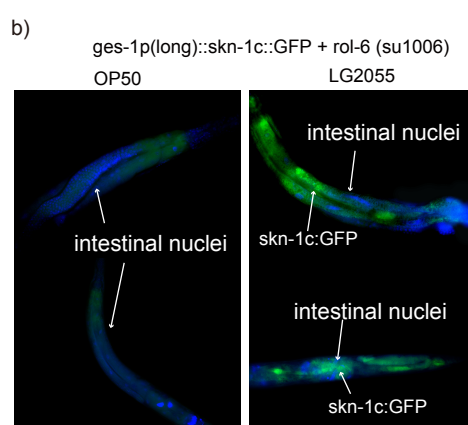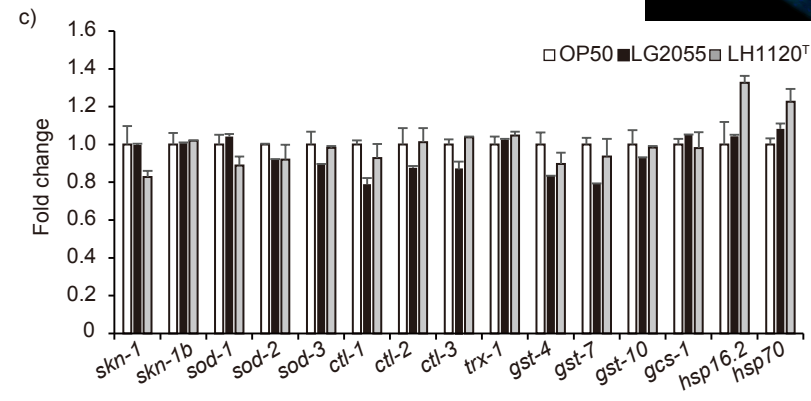

Supplement: Supplementary file 2 — Fig. S2 LG2055 feeding changed the gene expression of the molecules in p38MAPK signaling cascades. (a) Expression of the mRNA levels of the IIs pathway‐related genes, let‐363, and p38MAPK signaling molecules, on adult day 10 after OP50 or LG2055 feeding was measured by real‐time PCR. Expression levels of mRNA were normalized to the act‐1 mRNA expression level; error bars represent SEM. Differences compared with OP50‐fed worms were considered statically significant as *P < 0.05, *P < 0.01, and ***P < 0.001 by Student's t‐test. (b) Expression of skn‐1c was determined by fluorescence of GFP in the worms expressed from the gels10 transgenes after LG2055 or OP50 feeding for 10 days after feeding. (c) Expression level of SKN‐1 in the intestine of the worms was compared between LG2055 and OP50 feeding by the measurement of fluorescence intensity. Results are shown by the SEM of three independent experiments with 10 worms in each group; statistically evaluated by an unpaired Student's t‐test (*P < 0.05). [file ACEL-15-227-s002.pdf]
