# Supplementary material for: Effects and mechanisms of prolongevity induced by Lactobacillus gasseri SBT2055 in Caenorhabditis elegans
Source: Aging Cell. 2015 Dec 29;15(2):227–36. doi: 10.1111/acel.12431 (PMC4783334; doi:10.1111/acel.12431)
Supplement: Supplementary file 3 — Table S1 The effects of environmental variables upon lifespan in the presence or absence of LG2055. [file ACEL-15-227-s003.docx]

Table. S1 The effects of environmental variables upon lifespan in the presence or absence of LG2055.

|  | strain | feed type | mean±SD | log rank test vs OP50 |
| --- | --- | --- | --- | --- |
| Test 1 | N2 bristol | OP50 | 16.30±5.456 |  |
|  |  | LG2055 | 22.12±7.523 | *p*<0.0001 |
|  |  | L.gasseri JCM1131T | 19.29±7.393 | *p*<0.0001 |
|  |  | L.helveticus JCM1120T | 16.82±5.856 | N.S. |
| Test 2 | N2 bristol | OP50 | 14.90±5.114 |  |
|  |  | LG2055 | 21.47±8.151 | *p*<0.0001 |
|  |  | L.gasseri JCM1131T | 17.75±6.412 | *p*<0.0001 |
|  |  | L.helveticus JCM1120T | 15.32±5.829 | N.S. |
| Test 3 | N2 bristol | OP50 | 17.79±4.951 |  |
|  |  | LG2055 | 23.32±7.118 | *p*<0.0001 |
|  |  | L.gasseri JCM1131T | 20.18±5.811 | *p*<0.0001 |
|  |  | L.helveticus JCM1120T | 18.96±4.950 | N.S. |
| Total | N2 bristol | OP50 | 16.33±5.294 |  |
|  |  | LG2055 | 22.30±7.762 | *p*<0.0001 |
|  |  | L.gasseri JCM1131T | 19.07±6.626 | *p*<0.0001 |
|  |  | L.helveticus JCM1120T | 17.03±5.741 | N.S. |
| Test 1 | N2 bristol | UV-killed OP50 | 21.24±6.619 |  |
|  |  | UV-killed LG2055 | 27.83±7.422 | *p*<0.0001 |
| Test 2 | N2 bristol | UV-killed OP50 | 22.29±4.653 |  |
|  |  | UV-killed LG2055 | 28.39±3.162 | *p*<0.0001 |
| Total | N2 bristol | UV-killed OP50 | 22.73±5.369 |  |
|  |  | UV-killed LG2055 | 28.01±5.936 | p<0.0001 |
| Test 1 | N2 bristol | OP50 100% : LG2055 0% | 14.16±5.486 |  |
|  |  | OP50 75% : LG2055 25% | 16.09±6.205 | p<0.001 |
|  |  | OP50 50% : LG2055 50% | 17.15±6.317 | p<0.0001 |
|  |  | OP50 25% : LG2055 75% | 18.28±6.679 | p<0.0001 |
|  |  | OP50 0%: LG2055 100% | 21.73±5.322 | p<0.0001 |
| Test 2 | N2 bristol | OP50 100% : LG2055 0% | 13.81±3.182 |  |
|  |  | OP50 75% : LG2055 25% | 17.11±4.896 | p<0.001 |
|  |  | OP50 50% : LG2055 50% | 17.64±2.785 | p<0.0001 |
|  |  | OP50 25% : LG2055 75% | 18.91±6.111 | p<0.0001 |
|  |  | OP50 0%: LG2055 100% | 21.12±4.261 | p<0.0001 |
| Total | N2 bristol | OP50 100% : LG2055 0% | 13.87±4.921 |  |
|  |  | OP50 75% : LG2055 25% | 16.56±5.877 | p<0.001 |
|  |  | OP50 50% : LG2055 50% | 17.33±6.317 | p<0.0001 |
|  |  | OP50 25% : LG2055 75% | 18.68±6.679 | p<0.0001 |
|  |  | OP50 0%: LG2055 100% | 21.44±5.322 | p<0.0001 |
| Test 1 35˚C | N2 bristol | OP50 | 9.24±7.472 |  |
|  |  | LG2055 | 12.03±7.126 | *p*<0.001 |
| Test 2 35˚C | N2 bristol | OP50 | 10.18±4.937 |  |
|  |  | LG2055 | 13.06±7.836 | *p*<0.001 |
| Total 35˚C | N2 bristol | OP50 | 9.88±6.155 |  |
|  |  | LG2055 | 12.59±7.422 | p<0.001 |
| Test 1 | *mev-1*  (kn-1) | OP50 | 10.12±4.874 |  |
|  |  | LG2055 | 14.98±4.945 | *p*<0.0001 |
| Test 2 | *mev-1* (kn-1) | OP50 | 11.65±5.318 |  |
|  |  | LG2055 | 15.17±4.365 |  |
| Test 3 | *mev-1* (kn-1) | OP50 | 11.07±4.267 |  |
|  |  | LG2055 | 15.52±3.711 | *p*<0.0001 |
| Total | *mev-1* (kn-1) | OP50 | 10.91±4.472 |  |
|  |  | LG2055 | 15.22±4.755 | p<0.0001 |

*p<0.05, *p<0.01, ***p<0.001, log-rank, Data are obtained by three independent experiments with 100 worms grown at 20 °C per group. Lifespan survival statistics are in Table.S1. Thermotolerance was measured at 35 °C.
