# Supplementary material for: Effects and mechanisms of prolongevity induced by Lactobacillus gasseri SBT2055 in Caenorhabditis elegans
Source: Aging Cell. 2015 Dec 29;15(2):227–36. doi: 10.1111/acel.12431 (PMC4783334; doi:10.1111/acel.12431)
Supplement: Supplementary file 4 — Table S2 The effects of genetic variables upon lifespan in the presence or absence of LG2055. [file ACEL-15-227-s004.docx]

Table. S2 The effects of genetic variables upon lifespan in the presence or absence of LG2055.

|  | strain | feed type | mean±SD | log rank test vs OP50 |
| --- | --- | --- | --- | --- |
| Test 1 | *daf-2 (e1368)* | OP50 | 18.31±2.556 |  |
|  |  | LG2055 | 22.27±7.523 | p<0.001 |
| Test 2 | *daf-2 (e1368)* | OP50 | 18.69±5.452 |  |
|  |  | LG2055 | 23.12±4.391 | p<0.001 |
| Test 3 | *daf-2 (e1368)* | OP50 | 17.11±5.456 |  |
|  |  | LG2055 | 22.63±3.362 | p<0.001 |
| Total | *daf-2 (e1368)* | OP50 | 17.72±3.952 |  |
|  |  | LG2055 | 22.67±5.141 | p<0.001 |
| Test 1 | *daf-16 (mgDf50)* | OP50 | 11.61±5.456 |  |
|  |  | LG2055 | 15.02±3.961 | p<0.001 |
| Test 2 | *daf-16 (mgDf51)* | OP50 | 10.89±3.627 |  |
|  |  | LG2055 | 14.94±4.593 | p<0.001 |
| Test 3 | *daf-16 (mgDf52)* | OP50 | 12.07±5.251 |  |
|  |  | LG2055 | 15.82±6.732 | p<0.001 |
| Total | *daf-16 (mgDf50)* | OP50 | 11.72±4.892 |  |
|  |  | LG2055 | 15.28±4.556 | p<0.001 |
| Test 1 | *skn-1 (zu135)* | OP50 | 15.25±3.999 |  |
|  |  | LG2055 | 15.80±3.717 | N.S. |
| Test 2 | *skn-1 (zu135)* | OP50 | 14.67±3.762 |  |
|  |  | LG2055 | 14.40±3.980 | N.S. |
| Test 3 | *skn-1 (zu135)* | OP50 | 16.82±2.197 |  |
|  |  | LG2055 | 16.79±4.134 | N.S. |
| Total | *skn-1 (zu135)* | OP50 | 15.57±4.718 |  |
|  |  | LG2055 | 15.68±5.211 | N.S. |
| Test 1 | *skn-1 (zu67)* | OP50 | 12.51±3.120 |  |
|  |  | LG2055 | 13.25±2.975 | N.S. |
| Test 2 | *skn-1 (zu67)* | OP50 | 12.50±2.724 |  |
|  |  | LG2055 | 13.26±3.100 | N.S. |
| Test 3 | *skn-1 (zu67)* | OP50 | 13.16±2.999 |  |
|  |  | LG2055 | 13.95±2.907 | N.S. |
| Total | *skn-1 (zu67)* | OP50 | 12.99±2.173 |  |
|  |  | LG2055 | 13.25±3.521 | N.S. |
| Test 1 | *tir-1 (ok1052)III* | OP50 | 12.33±3.063 |  |
|  |  | LG2055 | 14.60±2.918 | *p<0.05* |
| Test 2 | *tir-1 (ok1052)* | OP50 | 11.83±3.971 |  |
|  |  | LG2055 | 15.00±4.028 | *p<0.05* |
| Test 3 | *tir-1 (ok1052)* | OP50 | 10.50±4.861 |  |
|  |  | LG2055 | 16.33±4.850 | *p<0.05* |
| Total | *tir-1 (ok1052)* | OP50 | 11.87±4.287 |  |
|  |  | LG2055 | 15.05±3.771 | *p<0.01* |
| Test 1 | *nsy-1 (ag3)* | OP50 | 17.63±5.782 |  |
|  |  | LG2055 | 17.25±5.196 | N.S. |
| Test 2 | *nsy-1 (ag3)* | OP50 | 14.40±3.717 |  |
|  |  | LG2055 | 15.50±4.170 | N.S. |
| Test 3 | *nsy-1 (ag3)* | OP50 | 15.78±4.817 |  |
|  |  | LG2055 | 16.17±4.641 | N.S. |
| Total | *nsy-1 (ag3)* | OP50 | 16.03±4.671 |  |
|  |  | LG2055 | 16.28±5.544 | N.S. |
| Test 1 | *sek-1 (km4)* | OP50 | 11.50±7.306 |  |
|  |  | LG2055 | 12.50±4.392 | N.S. |
| Test 2 | *sek-1 (km4)* | OP50 | 13.54±3.076 |  |
|  |  | LG2055 | 13.56±3.859 | N.S. |
| Test 3 | *sek-1 (km4)* | OP50 | 12.57±3.458 |  |
|  |  | LG2055 | 13.03±3.770 | N.S. |
| Total | *sek-1 (km4)* | OP50 | 12.81±5.829 |  |
|  |  | LG2055 | 13.30±3.761 | N.S. |
| Test 1 | *pmk-1 (km25)* | OP50 | 13.65±5.456 |  |
|  |  | LG2055 | 13.95±5.104 | N.S. |
| Test 2 | *pmk-1 (km25)* | OP50 | 13.98±5.791 |  |
|  |  | LG2055 | 14.20±5.187 | N.S. |
| Test 3 | *pmk-1 (km25)* | OP50 | 13.80±5.440 |  |
|  |  | LG2055 | 14.27±5.631 | N.S. |
| Total | *pmk-1 (km25)* | OP50 | 13.84±5.194 |  |
|  |  | LG2055 | 14.12±5.292 | N.S. |

Data were analyzed using the independent Log rank test. Data were obtained by three independent experiments with 40 worms grown at 20 °C per group.
