# Supplementary material for: Effects and mechanisms of prolongevity induced by Lactobacillus gasseri SBT2055 in Caenorhabditis elegans
Source: Aging Cell. 2015 Dec 29;15(2):227–36. doi: 10.1111/acel.12431 (PMC4783334; doi:10.1111/acel.12431)
Supplement: Supplementary file 5 — Table S3 Primer sets used for quantitative RT‐PCR. [file ACEL-15-227-s005.docx]

Table S3. Primer Sets Used for Quantitative RT-PCR

| **Gene Name** | **Primer** | **Sequence** |
| --- | --- | --- |
| *skn-1* | forward | 5'-CTCTCTTCTGGCATCCTCTACCA-3' |
|  | reverse | 5'-TTCTTGGATTCTTCTTCTTGTTCGT-3' |
| *skn-1b* | forward | 5'-GGCAGCAACCTTGTTCTTTC-3' |
|  | reverse | 5'-GGACGTCAACAGCAGACTCA-3' |
| *ctl-1* | forward | 5'-GCGGATACCGTACTCGTGAT-3' |
|  | reverse | 5'-GTGGCTGCTCGTAGTTGTGA-3' |
| *ctl-2* | forward | 5'-TCCGTGACCCTATCCACTTC-3' |
|  | reverse | 5'-TGGGATCCGTATCCATTCAT-3' |
| *ctl-3* | forward | 5'-GCGGATACCGTACTCGTGAT-3' |
|  | reverse | 5'-GTGGCTGCTCGTAGTTGTGA-3' |
| *gst-4* | forward | 5'-GATGCTCGTGCTCTTGCTG-3' |
|  | reverse | 5'-CCGAATTGTTCTCCATCGAC-3' |
| *gst-7* | forward | 5'-GGACAAGACTTCGAGGACAAC-3' |
|  | reverse | 5'-AACTGACGAGCCAAGTAACG-3' |
| *gst-10* | forward | 5'-AAGAGATTGTGCAGACTGGAG-3' |
|  | reverse | 5'-AGAACATGTCGAGGAAGGTTG-3' |
| *sod-1* | forward | 5'-CGTAGGCGATCTAGGAAATGTG-3' |
|  | reverse | 5'-AACAACCATAGATCGGCCAACG-3' |
| *sod-2* | forward | 5'-CTTCAAAACACCGTTCGCTG-3' |
|  | reverse | 5'-CAGTGGAACAAGTCCAGTT-3' |
| *sod-3* | forward | 5'-TTCAAAGGAGCTGATGGACACT-3' |
|  | reverse | 5'-AAGTGGGACCATTCCTTCCAA-3' |
| *trx-1* | forward | 5'-TCCAACACTTTTTGACGCAG-3' |
|  | reverse | 5'-CAAGATGATGCCGACTTTCA-3' |
| *clk-1* | forward | 5'-GCAATAGCTCCCTTGCATCC-3' |
|  | reverse | 5'-AGCACATACTGCTGCTTCTC-3' |
| *hsp-16.2* | forward | 5'-CTATTTCCGTCCAGCTCAAC-3' |
|  | reverse | 5'-TTTGTTCAACGGGCGCTTGC-3' |
| *hsp-70* | forward | 5'-ACGGGATGCTGTCATTACTG-3' |
|  | reverse | 5'-ATGTACCTCCTCCCAAATCG-3' |
| *gcs-1* | forward | 5'-ATTTCTGGAGCATCTGGTGG-3' |
|  | reverse | 5'-AAGGTAGTCCGTTGACGTGG-3' |
| *daf-2* | forward | 5'-AAAAGATTTGGCTGGTCAGAGA-3' |
|  | reverse | 5'-TTTCAGTACAAATGAGATTGTCAGC-3' |
| *daf-16* | forward | 5'-TTCAATGCAAGGAGCATTTG-3' |
|  | reverse | 5'-AGCTGGAGAAACACGAGACG-3' |
| *age-1* | forward | 5'-TTCGTAGAGTGGTTTTGGGC-3' |
|  | reverse | 5'-GGTTTCTTCAATTGGCTGGA-3' |
| *let-363* | forward | 5'-TCTCCGCTCCAGCTACAAAT-3' |
|  | reverse | 5'-AATTCGGCTCCGATATTGTG-3' |
| *wdr-23* | forward | 5'-AAATGGAACGAAAATCGTGG-3' |
|  | reverse | 5'-TCAAGAATCGACCAACCACA-3' |
| *aak-2* | forward | 5'-TGCTTCACCATATGCTCTGC-3' |
|  | reverse | 5'-GGGAACAAGTAGTTCGGCAA-3' |
| *par-4* | forward | 5'-ACTTTAACGAGCGTCCAACG-3' |
|  | reverse | 5'-CTCCTGTTCTCATTCGCTCC-3' |
| *unc-43* | forward | 5'-CTTGCAATCGAAGTGAACGA-3' |
|  | reverse | 5'-AAACGGTGTTGGTCCTCATC-3' |
| *sek-1* | forward | 5'-GCCGATGGAAAGTGGTTTTA-3' |
|  | reverse | 5'-TAAACGGCATCGCCAATAAT-3' |
| *nsy-1* | forward | 5'-AGTTGTAGACACGCCGGTTA-3' |
|  | reverse | 5'-TGCACTGTTCAGGCTTTCAC-3' |
| *pmk-1* | forward | 5'-CCGACTCCACGAGAAGGATA-3' |
|  | reverse | 5'-AGCGAGTACATTCAGCAGCA-3' |
| *act-1* | forward | 5'-GCTGGACGTGATCTTACTGATTACC-3' |
|  | reverse | 5'-GTAGCAGAGCTTCTCCTTGATGTC-3' |
