# Supplementary material for: Effects and mechanisms of prolongevity induced by Lactobacillus gasseri SBT2055 in Caenorhabditis elegans
Source: Aging Cell. 2015 Dec 29;15(2):227–36. doi: 10.1111/acel.12431 (PMC4783334; doi:10.1111/acel.12431)
Supplement: Supplementary file 6 — Appendix S1 Supporting experimental procedures. [file ACEL-15-227-s006.docx]

**Supporting experimental procedures**

**Preparation of LG2055 cells**

*Lactobacillus gasseri* SBT2055 (LG2055) was originally isolated by Milk Science Research Institute, Megmilk Snow Brand Co., Ltd. (Tokyo, Japan) and deposited in the International Patent Organism Depository, National Institute of Advanced Industrial Science and Technology (Tsukuba, Ibaraki 305-8566, Japan). This strain was selected on the basis of their human origin, nonpathogenic status, resistance to intestinal acid and bile, and previously proven to survive transiently through the GI tract in human studies and to be free of side effects (Fujiwara *et al.* 2001). The LG2055 was inoculated (1 %) into deMan, Rogosa and Sharpe (MRS) broth (Becton–Dickinson and Company, MD, USA) and incubated for 18 h at 37 °C. Then, LG2055 cells were collected by centrifugation at 5,000 x g for 10 min at 4 °C. Cells were washed twice with PBS. The washed LG2055 cells were resuspended in 10 times the volume of B broth. The suspended cells were spread on NGM plate containing 5-fluorouracil (Wako, Osaka, Japan). Dried up 2 hours. The ability of other strains of *Lactobacillus* spp., which were *Lactobacillus gasseri* JCM1131^T^, *Lactobacillus helveticus* JCM1120^T^ to affect longevity and aging were examined for comparison with that of LG2055. The cells of these strains were prepared by the same methods used to prepare the LG2055 cells.

**Ingestion of FITC labeled LG2055**

FITC-labeling was accomplished by incubating approximately 100ul of a LG2055 cell pellet in 1ml PBS containing 1mg FITC. The solution was centrifuged to repellet the cell, which were then washed 3times in PBS. Pellet were suspend in M9 buffer and spread on NGM plate. Young adult stage worms were placed on FITC-labeled LG2055 NGM plate. After Twenty minutes, fluorescence was examined microscopically.

**Age synchronization**

Egg-bearing worms were collected from NGM plates with OP50. Eggs were extracted in the cleaning solution (0.7 m NaOH with 2% Na-hypochlorite) and then washed with M9 buffer at least three times. Eggs were transferred to OP50 plate and incubated overnight at 20 °C with fairly vigorous shaking to obtain synchronous L1 animals.

**Motility Assays**

Single animals were maintained on individual NGM plates throughout adulthood and were transferred to fresh plates seeded with OP50 bacteria before locomotion or pumping rates were determined. Locomotion rates were determined by counting body bends per 1min of animals moving on the NGM plate using a dissecting microscope. Pumping rates were assayed by counting the number of movements per 30s of the rear bulb of the pharynx of animals within the bacterial lawn using a dissecting microscope.

**Age pigment (Lipofuscin) Accumulation**

Days 10 after OP50 or LG2055 feeding, worms were transferred onto 2% (w/v) agarose pads for microscopy. We captured images on a BZ-9000 (KEYENCE, Osaka, Japan). Lipofuscin levels were measured using ImageJ software (NIH Image) by determining the average pixel intensity in each worm body.

**Oil Red O staining**

Oil-Red-O staining was conducted by washing 50-100 day-12 adult worms with 1x PBS. Worms were washed three times with PBS and allowed to settle by gravity. To permeabilize the cuticle, worms were resuspended in PBS containing 2% paraformaldehyde. Samples were gently rocked for 1h at room temperature. Animals were allowed to settle by gravity, buffer was aspirated, and worms were washed with 1x PBS to remove PFA. Worms were then resuspended in 1x PBS 60% isopropanol 0.01% triton X-100 and incubated for 15 minutes. Oil-Red-O is prepared as follows: a 0.3g/100mL isopropanol stock solution equilibrated for several days was freshly diluted to 60% with water and rocked for at least 1h, then filtered with 0.22μm-filter. After allowing worms to settle, isopropanol was removed, 1 mL of 60% Oil-Red-O stain was added, and animals were incubated overnight with rocking. Dye was removed after allowing worms to settle, and 200 μL of 1x PBS 0.01% Triton X-100 was added. Fat content was monitored and imaged by KEYENCE BZ-9000. Images were inverted to give a dark background and the blue plane of each RGB image was top-hat filtered to compensate for non-uniform background, then auto-threshold to identify regions corresponding to worms. Within these regions, the level of Oil-Red-O was quantified from the original images by determining the excess intensity in the red channel in comparison to the blue and green channels, with regions with less red than blue or green ignored. These red-excess regions were auto-threshold to separate background redness from Oil-Red-O stained areas, with mean fatness per image estimated as the total intensity within stained regions normalized by the area of the worm regions.

**Senescence-associated β-galactosidase activity assay**

Senescence-associated β-galactosidase (SA-β-gal) activity was measured with β-Galactosidase Staining Kit (BioVision, CA, USA). The protocol of the manufacturer’s instructions was described modified for *C.elegans*. 17 days old worms were washed in M9 buffer, fixed for 30 min at room temperature with 0.5 ml of Fixative Solution, washed and incubated overnight at 37 °C with the Staining Solution Mix. Bodies were observed under a microscope for development of blue color. We captured images on a BZ-9000 (KEYENCE, Osaka, Japan). Staining area were measured using ImageJ software (NIH Image) by determining the average pixel intensity in each worm body.

**Thermotolerance**

Assays of stress resistance were performed essentially as described in (Yanase *et al.* 2002). Thermotolerance was measured using adult day 10 worms at 35 °C. Age-synchronous worms were grown to the L4/ young adult stage on NGM plate seeded with OP50, then transferred individually onto the OP50 or LG2055 plate and maintained at 20°C. At the start of the thermotolerance assay, 100 worms from each feeding groups were transferred to a fresh NGM plate with OP50, and shifted to 35˚C. Numbers of surviving and dead animals were scored every 2 h. The statistical software used was EZR, which also computed the median lifespan.

**Oxidative Stress Assay**

Worms were transferred as late L4 stage or young adults to NGM plates containing 25 mM paraquat (Wako, Osaka, Japan) and scored for survival every 2 hr.

***skn-1*:GFP localization assay**

LG349 geIs10 (ges-1p(long)::skn-1c::GFP + rol-6 (su1006)). Age-synchronous worms were growth to the L4/ young adult stage on NGM plates seeded with OP50, then transferred individually into the OP50 or LG2055 plates and maintained at 20°C. After 10 days, the worms were washed 3 times.The resulting fluorescence was quantitated using BZ-9000 (KEYENCE, Osaka, Japan).

**RNA preparation and Q-PCR analysis**

Total RNA was isolated from 500 worms using the RNeasy plus Mini Kit (QIAGEN, Venlo, Netherlands). Reverse transcriptase (Life technologies,) was used for oligo (dT) primed first-strand cDNA synthesis. The qPCR analysis was carried out on an ABI PRISMR 7000 Sequence Detection Systems (Life technologies) with SYBR green (Life technologies). The ΔΔCt method was used to quantify the amount of the mRNA level relative to that of *act-1*. The primers for Q-PCR were designed using primer3.

**Western blot analysis.**

For western blots, N2 (wild type) worms were collected by centrifugation. Worm pellets were washed and grind. Approximately 30 ml of pellets were boiled in 5 volumes sample buffer for 5 min. Different aliquots of extracted proteins were resolved on 12.5% acrylamide SDS-PAGE gels. Proteins were electrophoretically transferred to nitrocellulose membranes and membranes were probed with anti-skn-1 antibody (Santa Cruz Biotechnology) and anti phospho-p38MAPK antibody (Cell signaling technologies). HRP signals were visualized with Immobilon Western chemiluminescent HRP substrate (Millipore) and LAS-4000 image analyzer (Fujifilm, Tokyo, Japan). Band intensities were quantified by ImageJ software (National Institutes of Health, MD, USA).

**SOD activity assays**

SOD activity was measured using the Oxyselect superoxide dismutase activity assay (Cell Biolabs, San Diego, CA, USA) involving the inhibition of superoxide-induced chromogen chemiluminescence by SOD, according to the manufacturer's instructions. For the measurement of the SOD activity, protein extracts were pretreated with 5 mM NaCN to inhibit Cu/Zn-SOD activity. The absorbance of each well was read using a BioTek synergy4 multi-mode microplate reader (BioTek Instruments, Inc. Winooski, VT, USA) using 490 nm as the primary wavelength. At least three independent experiments were performed for each assay.

**GSH/GSSG Assay**

Reduced glutathione (Li *et al.*), oxidized glutathione (GSSG), and GSH/GSSG ratio were measured with a Glutathione Assay Kit (Promega, Tokyo, Japan). This kit can determine GSH and GSSG individually. The GSH and GSSG levels were calculated according to the standard curve, and then the GSH/GSSG ratio was calculated. Here, 50 worms each group were placed in wells of 96-well white plates. Total glutathione lysis reagent or Oxidized glutathione lysis reagent were added to the wells containing the worms. The plates were shaken and Luciferin generation reagent added. Total glutathione luminescence was measured using BioTek synergy4.Data analysis based on calculating the ratio from the GSH standard curves.

**Dehydroethidium (DHE) Staining**

Twenty of adult day 10 worms were washed 3 times in M9 buffer, incubated with 3 μmol/l of DHE in M9 buffer for 4hr, and analyzed for fluorescence intensity by confocal microscopy. Raw data from confocal microscopy were then analyzed using ImageJ.

**Mitochondria staining**

Mitotracker-CMX Ros (Invitrogen) were dissolved in DMSO, diluted in 1xPBS (final concentration of 400nM). After the worms were fed on OP50 or LG2055 for 10days, they were washed three times with PBS and allowed to settle by gravity. To permeabilize the cuticle, worms were resuspended in PBS containing 2% paraformaldehyde. Worms were gently rocked for 1h at room temperature. Animals were allowed to settle by gravity, then resuspended in 1x PBS 60% isopropanol 0.01% Triton X-100 and incubated for 15 minutes. After allowing worms to settle, isopropanol was removed, 1 mL of Mitotracker-CMX Ros solution was added, and animals were incubated overnight with rocking. Dye was removed after allowing worms to settle, and 200 μl of 1x PBS 0.01% Triton X-100 was added. Mitochondria levels were monitored by fluorescence microscopy. The fluorescence density was measured using ImageJ software.

**JC-1 Staining**

Nematodes, 10 days from young adult were collected and washed three times with S basal buffer. Worms were resuspended in PBS containing 2% paraformaldehyde. Sample were transferred to microtubes containing 1 x JC-1 reagent, and incubated at 37°C in a 5% CO2 incubator for 30 minutes. Sample were washed and suspended in Assay buffer, placed on 96 well black plates. Measurements of red fluorescence (excitation 550 nm, emission 600 nm) and green fluorescence (excitation 485 nm, emission 535 nm) used a fluorescence plate reader BioTek synergy4.

**Measurement of ATP levels**

About 100 10–day or 15-day fed worms (post-larval L4 stage) were collected, washed four times with M9 buffer, boiled for 20 minutes and quickly frozen in −80°C. A Roche ATP Bioluminescent HSII kit was used to measure the ATP concentrations, which measures bioluminescence emitted by the ATP-dependent oxidation of D-luciferin catalyzed by luciferase. The ATP concentrations were determined using a standard curve derived from the bioluminescence of known ATP concentrations (HSII kit). BioTek synergy4 was used to measure the levels of bioluminescence. Protein concentrations were measured using the BCA assay. Results were expressed as amounts of ATP nmol/mg of proteins. Assaying was repeated in triplicate, and the average ATP concentrations and SD were calculated.

**Statistical analysis**

Data were analyzed using the independent Log rank test, Student's t-test, ANOVA, and the Bonferroni test for multiple comparisons of means with SPSS statistics software (IBM, USA) and EZR (Saitama Medical Center, Jichi Medical University), which is a graphical user interface for R (The R Foundation for Statistical Computing, version 1.21).
